# General practitioners with a special interest in respiratory medicine: national survey of UK primary care organisations

**DOI:** 10.1186/1472-6963-5-40

**Published:** 2005-05-27

**Authors:** Hilary Pinnock, Gopalakrishnan Netuveli, David Price, Aziz Sheikh

**Affiliations:** 1 Division of Community Health Sciences: GP Section, University of Edinburgh, 20, West Richmond St, Edinburgh, Scotland. EH8 9DX. UK; 2 Department of Primary Care and Social Medicine, 3rd Floor, The Reynolds Building, St Dunstan's Road, London, England. W6 8RP. UK; 3 Department of General Practice and Primary Care, University of Aberdeen, Foresterhill Health Centre, Westburn Road, Aberdeen, Scotland. AB25 2AY. UK

## Abstract

**Background:**

To meet the universally recognised challenge of caring for people with long-term diseases many healthcare cultures are encouraging family physicians to develop specialist skills. We aimed to determine the major factors influencing the appointment of respiratory General Practitioners with a Special Interest (GPwSI) in the UK, and to determine the priority attached to the potential roles, perceived barriers to implementation, and monitoring planned.

**Methods:**

We sent a piloted semi-structured questionnaire to a random sample of 50% of English and Welsh primary care organisations (PCOs) (n = 161) during winter 2003. In addition to descriptive statistics, we used hierarchical cluster analysis to classify service priorities. Free-text responses to open-ended questions were analysed qualitatively by a multidisciplinary group to identify emerging themes.

**Results:**

Of the 111 (69%) PCOs who responded, 7 (6%) already have, and a further 35 (32%) are planning, a respiratory GPwSI service. This proportion is considerably lower than in specialities linked to National Health Service clinical priorities. Local needs and pressure on hospital beds were the main described motives for developing a service. Stated service priorities were to relieve pressure on secondary care and to improve quality of care, including the strategic planning of respiratory services within PCOs.

**Conclusion:**

The relatively few respiratory GPwSIs currently in post reflects the lack of government prioritisation of respiratory care. However, respiratory GPwSI services are increasingly being considered as a local strategy for reducing pressure on secondary care respiratory services and raising standards of chronic disease management in primary care.

## Background

The care of people with long-term disease is universally recognised as a major challenge, and national healthcare services around the world are reconfiguring to meet the demand [1,2]. Chronic respiratory disease is projected to rank as the fifth leading cause of morbidity by 2020 [3]. Increasingly, specialist roles are being devolved to family physicians, echoing recent global recognition of the contribution of primary care expertise to the management of common conditions such as respiratory disease [4]. Within the UK, the strong links between community-based practitioners and hospital specialists have long been valued and services increasingly draw on the resources of the two traditions to the mutual advantage of patients and clinicians [5].

General Practitioners with a Special Interest (GPwSIs), a key component of the UK National Health Service modernisation agenda [6], challenge traditional models of specialist care. The key policy driver is the imperative to reduce waiting lists for specialist opinions in areas such as ophthalmology, orthopaedics, dermatology, ear nose and throat surgery, and for specific procedures such as endoscopy [6,7]. The emphasis is on maintaining a family care perspective while developing defined specialist competencies to meet local healthcare need [7-9]. Primary care interest societies have broadened the potential remit of GPwSIs, delineating roles in a wider range of clinical areas and involving a more strategic role than was originally envisaged [10,11].

Despite being responsible for nearly a third of general practice consultations [12], one in eight emergency hospital admissions [13], and the major contributory factor in the winter bed crises [14], respiratory disease did not feature in any of the official documents [6-9]. This lack of national prioritisation of respiratory care is reflected internationally [15,16], with notable exceptions such as Finland and Australia [17]. The General Practice Airways Group (a UK charity focusing on delivering optimal respiratory care in community settings) considered this issue in a discussion paper outlining a number of potential roles for a respiratory GPwSI: leading the strategic planning from a primary care perspective, setting quality standards for respiratory care and providing clinical expertise for conditions most common in general practice [10]. These concepts, now embodied in a guideline [18], establish the potential of a respiratory GPwSI service to address many of the recognised unmet needs of people with respiratory and allergic conditions [19,20].

UK primary care organisations (PCOs) which manage care for populations of approximately 100,000, are charged with meeting the competing healthcare demands of their local community against a background of political pressures imposed by national policies and service frameworks. It is not known how many view positively the potential of respiratory GPwSIs. Our survey of primary care organisations in England and Wales aimed to determine the major factors influencing the appointment of respiratory GPwSI, and to determine the priority attached to potential roles, the perceived barriers to implementation of a GPwSI service and the monitoring planned.

## Methods

### Ethics

Grampian Research Ethics Committee advised that our survey of current practices and future plans did not require ethical approval. [MacLeod K, personal communication, July 2003]

### Questionnaire design

Our questions were based on a detailed review of the literature on the GPwSI initiative, and designed with the advice of health service administrators and clinicians with a specialist interest in primary care respiratory disease from all regions of the UK [21]. Minor adjustments were made after feedback from pilot PCOs. Closed questions included an option for adding additional responses; free-text comments were invited throughout. The relative importance of the potential issues that a respiratory GPwSI might address was assessed by asking respondents to rate priorities on a 5-point Likert-scale [5 = Top priority; 0 = No priority].

### Sampling procedures

During the winter of 2003/4 we sent the questionnaire to the chief executive of a 50% random sample (n = 161) of English and Welsh PCOs (Primary Care Trusts (PCTs) in England; Local Health Boards (LHBs) in Wales) asking them to forward the questionnaire to the person within their organisation best placed to complete the questionnaire. We phoned non-responding PCOs to identify the contact details of the person responsible for developing GPwSI services, to whom we e-mailed an electronic version of the questionnaire. Non-responders were sent a further postal reminder.

### Sample size calculation

To estimate the frequency of PCOs with an interest in appointing a respiratory GPwSI with 95% confidence, assuming an expected frequency of 10%, with a precision of 5%, we needed 108 usable responses. We anticipated a 70% response rate and therefore sampled 50% of the 322 PCOs in England and Wales.

### Data analysis

Responses to closed questions were treated as nominal data, whereas priority ratings were treated as on a linear scale. In addition to descriptive statistics, we looked for correlation between the different service priorities using Pearson correlation coefficients and used hierarchical cluster analysis to classify the priorities. All analyses were conducted using SPSS version 11.5.

Free-text responses to open-ended questions were thematically analysed by a multidisciplinary group involving practising and academic GPs, a health services manager, and a qualitative researcher. Using the principles of qualitative content analysis, we developed a coding frame and identified key emerging themes [22].

## Results

We received responses from 111/161 (69%) of the England and Welsh PCOs. Seven (6%) PCOs reported that they already had a respiratory GPwSI in post and a further 35 (32%) indicated that they were considering developing a respiratory GPwSI service.

### Comparison with other GPwSI services [Figure 1]

**Figure 1:**
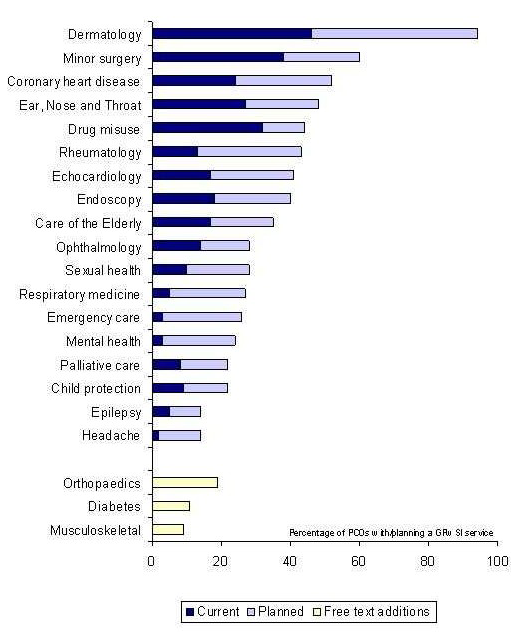
Current and planned GPwSI services

Ninety-eight PCOs gave information on existing GPwSIs or those under consideration in different specialities. Most commonly, PCOs tended to have three to five GPwSIs (46%) and a significant proportion (21%) had 10 or more. The top five GPwSIs in terms of frequency were respectively dermatology, minor surgery, coronary heart disease, ear, nose and throat surgery and drug misuse – areas highlighted by National Health Service policy [6,23]. Respiratory medicine ranked twelfth.

This early focus on nationally prioritised areas, with respiratory GPwSI services a later consideration, is reflected in the comments made by several PCOs:

*"The PCT *[Primary Care Trust]*has developed GPwSI led services for dermatology, ENT *[Ear, Nose and Throat], *orthopaedics and ophthalmology and is developing services for mental health, diabetes and emergency care in 2004/05. Following this, the PCT is considering the development of further GPwSI led services – including respiratory services" *(PCO-39: considering a respiratory GPwSI service)

*"Focusing on other areas of work at the moment. Principally the NHS *[National Health Service] *plan, cancer plan + NSFs *[National Service Frameworks]*" *(PCO-119: no plans for a respiratory GPwSI service)

### Major factors influencing the decision

Of the 42 English and Welsh PCOs who had, or were considering developing a respiratory GPwSI service, 33/42 (79%) stated that they were responding to local needs, often identified by audits of hospital activity:

*"Audit has shown that COPD *[chronic obstructive pulmonary disease]*/ asthma are causes of significant repeat admissions to hospital." *(PCO-54: considering a respiratory GPwSI service)

*"High level of deprivation and corresponding high levels of asthma morbidity. Have had asthma & respiratory as a local priority since ... late 90's." *(PCO-134: existing respiratory GPwSI service)

The prime motives for considering appointing a respiratory GPwSI are given in Table 1. Reducing pressure on secondary care, particularly admissions for chronic obstructive pulmonary disease, was the most commonly cited motive and improving chronic disease management in primary care was seen as a means to achieve this – possibly even diverting costs from in-patient to community care.

**Table 1:** Motivating factors and barriers identified by PCOs.

**Question**	**Options**	**N (%)**
**PCO-s that have (or are considering) a respiratory GPwSI service (n = 42)**		

What are the factors motivating your PCO to have/or consider a respiratory GPwSI service? (n = 41)*	Identified local needs	33 (80)
	Winter pressures on hospital beds	19 (46)
	New GP contract targets	13 (32)
	Government directive	5 (12)
	Influence of local personalities	5 (12)
	Drug cost containment	4 (10)
	Secondary care initiative	4 (10)
	Local patient pressure	1 (2)
	There's a pot of money available	1 (2)
	Pharmaceutical company influence	0

What are the major problems you will have to overcome? (n = 39)*	Competition with other local priorities	25 (64)
	Inadequate funding for respiratory GPwSI	20 (51)
	Inadequate infra structure support funding	15 (38)
	Respiratory disease is not a national priority	8 (21)
	Lack of local interest/expertise from GPs	6 (15)

**PCOs that are not considering a respiratory GPwSI service (n = 69)**		

Why is a respiratory GPwSI service not a priority in your PCO? (n = 67)*	Lack of local interest/expertise from GPs	24 (36)
	We have a respiratory specialist nurse	24 (36)
	Inadequate funding for respiratory GPwSI	13 (19)
	Inadequate infra structure support funding	13 (19)
	Respiratory disease is not a local priority	10 (15)
	Lack of local patient pressure	7 (10)
	Respiratory disease is not a national priority	6 (9)
	Opposition from secondary care	1 (1)
	Winter pressures are not a problem locally	0

* Not all PCOs answered all the questions..

*"Likely to be a growth area in our PCT in terms of better care leading to reduced admissions" *(PCO-72: considering a respiratory GPwSI service)

*"Improve appropriateness of secondary care referrals" *(PCO-127: considering a respiratory GPwSI service)

*"Funding has been an issue – we have always been keen. Now identified reducing hospital admissions as a way of freeing up funds." *(PCO-85: considering a respiratory GPwSI service)

The inclusion of respiratory targets in the General Medical Services contract for GPs [24] and the Primary Care Collaborative (a UK initiative to facilitate development in primary care) programme on chronic obstructive pulmonary disease [25] have given added impetus to the development of respiratory services.

*"We have become very motivated on this subject recently as we currently have 15 practices participating in Phase 3 of the Primary Care Collaborative. We are aware that we have neglected this area locally, as have many PCTs, however there is now real enthusiasm for change" *(PCO-33: no plans for a respiratory GPwSI service)

*"We do not currently have a GPwSI in respiratory service currently, but that does not mean we do not consider it as a priority. There is scope to develop this role to meet the requirements of nGMS *[new General Medical Services contract]" (PCO-7: no plans for a respiratory GPwSI service)

The 69 PCOs not planning a respiratory GPwSI service at this time cited local workforce issues as the main barrier [Table 1]: 24/69 (35%) felt that local GPs did not have the interest or necessary expertise to undertake the role whilst 24/69 (35%) already had a specialist respiratory nurse who was addressing the local needs appropriately. These issues echoed the problems that needed to be overcome by PCOs who were planning a service.

*"We do not have any GPs who presently have the skills (and just as importantly, the time) to give to this work. However we do have a specialist nurse led team working across primary and secondary care in respiratory illness." *(PCO-64: no plans for a respiratory GPwSI service)

*"We are currently recruiting for clinical leads in all areas – GP's w special interest in Respiratory medicine has not been filled" *(PCO-38: considering a respiratory GPwSI service)

Conversely, the presence of an 'existing respiratory champion' was a positive motivating factor.

*"Local need..... plus local GP with an interest" *(PCO-105: existing respiratory GPwSI service)

*"GP with expertise moved into area" *(PCO-85: considering a respiratory GPwSI service)

### The importance of specialist teams

The free text comments elaborated on the respective roles of GPwSIs and specialist nurses and emphasised the importance attached to team work, though the potential role of the GPwSI within that team varied.

*"However the LHB *[Local Health Board] *is in the process of implementing an integrated COPD team (with local trust) (Primary / Community care and secondary care). This team is lead by a consultant with links to GP's, Practice nurses, therapists and community nurses. The second phase will be to develop a GPwSI." *(PCO-158: considering a respiratory GPwSI service)

*"I think that for GPwSI's to be fully effective, robust support from respiratory nurses is essential. We have one F/T *[full-time]*lead nurse plus 2 P/T *[part-time] *Nurses" *(PCO-134: existing respiratory GPwSI service)

*"It is anticipated that the GPwSI role would support the provision of a nurse-led spirometry service" *(PCO-28: considering a respiratory GPwSI service)

### Priorities for a respiratory GPwSI service

In line with the factors motivating PCOs to develop respiratory GPwSI services, reducing hospital admissions was the top priority, with raising standards of respiratory care also highlighted. In contrast, allergy services were rarely prioritised [Table 2].

**Table 2:** Priorities for a GPwSI service. Based on the question "Please rate the priority of the following specific issues a respiratory GPwSI might address?" Score: 0 is no priority, 5 is top priority

**Priorities**		**Ratings**	
	N*	Mean	95% CI

Reducing acute respiratory admissions	38	4.5	4.3 to 4.7
Raising standards of respiratory care in practice	37	4.3	4.0 to 4.5
Reducing respiratory outpatient referrals	37	4.0	3.7 to 4.3
Strategic planning of respiratory services	34	3.9	3.6 to 4.3
Reducing respiratory outpatient referral waiting times	35	3.8	3.5 to 4.1
Improving access to spirometry	36	3.6	3.3 to 3.9
Coordination of General Medical Services quality framework for asthma and chronic obstructive pulmonary disease	34	3.6	3.4 to 3.9
More appropriate home oxygen use	33	3.2	3.0 to 3.5
Development of management templates/coordinated data collection and extraction	34	3.2	2.8 to 3,6
More appropriate home nebuliser use	32	3.2	2.9 to 3.5
Provision of an allergy service	30	1.9	1.7 to 2.2

* Not all PCOs answered all the questions

We found that some priorities tended to be highly correlated; for example PCOs prioritising reduction in the number of outpatient referrals also tended to prioritise reduction in outpatient waiting times (r = 0.8). Other highly correlated priorities were appropriate usage of home nebulisers and oxygen (r = 0.9), strategic planning of respiratory services and development of management templates/coordinated data collection and extraction (r = 0.8). Applying hierarchical cluster analysis, we were able to group the priorities into three main areas of consideration:

1) Relieving pressure on secondary care, including reducing admissions, outpatient referrals and waiting lists.

2) Improving quality of care, including strategic planning of respiratory services, raising standards of respiratory care in practice, coordination of GMS quality framework for asthma and chronic obstructive pulmonary disease and development of management templates/coordinated data collection and extraction. More specifically, improving access to spirometry and more appropriate home oxygen and nebuliser use also correlated with quality of care.

3) Providing allergy services.

### Infrastructure, support and monitoring planned

Monitoring focussed on the impact of a respiratory GPwSI service on secondary care, especially chronic obstructive pulmonary disease admissions, though nearly half the PCOs planned to assess patient satisfaction. Most PCOs appreciated the need to provide infra-structure support for a GPwSI and two thirds acknowledged the importance of supporting on-going professional development [Table 3].

**Table 3:** Infrastructure, support and monitoring planned for GPwSI service.

**Question**	**Options**	**N (%)**
What infra-structure/support have you got / do you plan for a respiratory GPwSI service? (n = 34)*	Clinical support – e.g. nurses, physiotherapists	30 (88)
	Medical equipment: e.g. spirometer, oximeter	23 (68)
	Training and on-going continuing professional development for the GPwSI	22 (65)
	Office support – e.g. room, desk, computer, etc.	17 (50)
	Administrative support – e.g. secretary	15 (44)

What monitoring are you undertaking/planning? (n = 38)*	Admission rates for chronic obstructive pulmonary disease	37 (97)
	Accident and emergency attendances	21 (57)
	Quality of respiratory care	19 (51)
	Practice prescribing for respiratory disease	19 (49)
	Patient satisfaction	19 (49)
	Admission rates for asthma	18 (46)
	Waiting times for chest clinic referrals	16 (43)
	Home oxygen use	5 (14)

* Not all PCOs answered all the questions

## Discussion

Although currently few in number, appointment of respiratory GPwSIs is currently being considered by nearly a third of PCOs in the UK: still however considerably less than in specialities prioritised by government policy. Reducing pressure on secondary care services is the key motive for appointing a respiratory GPwSI, a top priority for the role, and the aspect of the service most likely to be monitored. Improving the quality of respiratory care is also highlighted, both as a means of reducing referrals and admissions and also in line with the increasing emphasis on chronic disease management in primary care [24].

### Limitations of study

We achieved a response rate of 69% and our results may not reflect the situation in the non-responding PCOs. It is probable that PCOs most interested in appointing a respiratory GPwSI will have responded promptly; those who had not yet focussed on the potential of a respiratory GPwSI service may have been less motivated to respond, indeed in some organisations with few plans for GPwSI services it may not have been clear who would be best placed to answer the questionnaire. This would result in an overestimate of the interest in a respiratory GPwSI.

GPwSI services are a rapidly developing initiative and despite extensive background reading, wide consultation and piloting the questionnaire it is likely that the closed question format did not predict all possible responses. Each question, therefore, offered the opportunity to add additional responses. Reassuringly, for most questions the space was used to clarify the closed responses rather than add new options. The exception was the very wide range of innovative GPwSI services listed. Those offered most frequently have been included in Figure 1, though the frequency cannot be directly compared with those specialities for which a prompt was given.

The answers to closed questions and free text responses can only provide limited insight into the development of respiratory GPwSI services. We did not attempt to define a respiratory GPwSI because the absence (at the time of the survey) of agreed accreditation processes and the concept of a locally developed service would have made that difficult, so it is likely that there was some variation in the interpretation of the question. However, the responses do provide a quantitative assessment of the interest in this initiative, and the comments indicate the potential value of a follow-on in-depth exploration of perspectives on the development of respiratory GPwSI services.

### Main strengths of study

We achieved our anticipated response rate and therefore exceeded our intended sample size. Our piloted questionnaire appeared to be acceptable to PCOs and we identified no problems with completion. Our random sampling strategy should ensure national generalisability across England and Wales. We used an integrated quantitative and qualitative analytical paradigm, thus increasing the validity of our findings [26].

### Interpretation of findings in relation to previously published work

Primary care organisations in England and Wales have adopted the concept of GPwSIs initially focusing on areas driven by government policy [6,23], but increasingly as an option for developing a wider range of specialities in order to meet local needs. Our data suggest that, although only 6% of PCOs currently have a respiratory GPwSI in post, there may be welcome interest in respiratory disease with nearly a third of PCOs considering a respiratory GPwSI service.

In keeping with national policy [6], the priority for respiratory GPwSI services in most PCOs is to reduce pressure on secondary care. Admission rates are a key target, especially for chronic obstructive pulmonary disease where in-patient stays are often prolonged and an important factor in winter pressure on hospital beds [14]. GPwSIs may also contribute to local strategies designed to meet government targets for a 3.5% per year shift of outpatient consultations to primary care [27-29]. Experience from government-driven initiatives in Finland and Australia exemplify the importance of engaging primary care specialists as care is shifted to the community [17].

The increasing global emphasis on chronic disease management [1,2] and empowering patient self-management [30] may have influenced the priority attached to the strategic role, seen by many PCOs as potentially within the remit of a respiratory GPwSI. In the UK, the new primary care contract, with a focus on respiratory chronic disease provides an important context for this initiative [24]. Internationally, the challenge of managing long-term conditions may be an useful argument for both primary and secondary care specialists, campaigning nationally to encourage governments to prioritise respiratory care and locally to ensure that the needs of respiratory patients are met [17].

The lack of priority attached by PCOs to allergy services reflects the concern expressed by the House of Commons Health Committee report on the current under-provision of allergy services in the UK [31]. This report recommends the development of a cadre of GPwSIs to give focus and expertise to the treatment of allergy in primary care.

It is encouraging that two-thirds of the PCOs indicated that their planned infra-structure for a respiratory GPwSI service included support for on-going training and professional development; however, there should be concern that this was not universally prioritised. Agreed procedures for accrediting a respiratory GPwSI have now been agreed, and emphasise the importance of appropriate training, mentoring and accreditation required to assure quality [9,32]. Concerns have already been expressed that the locally defined contracts could lead to unacceptable variations in the contractual obligations, remuneration and support [11]. Whilst diversity in locally defined roles is to be encouraged, training tailored to meet that role is a universal requirement [33].

Our survey focused specifically on the recently defined GPwSI initiative. However, there is already a strong tradition both in primary and secondary care of specialist respiratory nurses [34-36] with many well established teams leading, for example 'Hospital at Home' schemes [37]. Not surprisingly, therefore, nearly 90% of PCOs envisaged the development of specialist teams to support their planned GPwSI and a third of PCOs in our survey indicated that they either had or were considering a specialist nurse rather than a GPwSI appointment. Local factors, such as availability of GP or nurse expertise, were important in determining the planned workforce configuration.

## Conclusion

Healthcare organisations in the UK are responding positively to the challenge of reconfiguring the workforce to meet local needs. Although the initial focus has been on areas highlighted by National Health Service policy, respiratory GPwSIs are increasingly being considered, both as a means of reducing pressure on secondary care, and also raising standards in primary care to meet the challenge of chronic disease management.

## Abbreviations and explanation of terms

COPD Chronic obstructive pulmonary disease

ENT Ear. Nose and Throat surgery.

GMS General Medical Services. The GMS contract, recently updated as the 'new' GMS contract (nGMS), governs provision of primary care services.

GPwSI General Practitioners with a Special Interest.

LHB Local Health Board

PCO Primary Care Organisations. These organisations, known as PCTs in England and LHBs in Wales, commission local healthcare services.

PCT Primary Care Trust

NHS National Health Service

NSFs National Service Frameworks. These NHS documents set national standards for the provision of care for a range of disease areas.

Primary Care Collaborative is a UK initiative to facilitate development in primary care. Phase 3 of this initiative includes a focus on chronic obstructive pulmonary disease.

## Competing interests

The author(s) declare that they have no competing interests.

## Authors' contributions

HP initiated the idea for the study and with AS led the development of the protocol, securing of funding, study administration, data analysis, interpretation of results and writing of the paper. GN provided statistical advice and DP provided advice on the development of the protocol and the interpretation of results. All authors reviewed the final manuscript. HP and AS are study guarantors.

## Funding

General Practice Airways Group.

## Pre-publication history

The pre-publication history for this paper can be accessed here:



## References

[B1] World Health Organisation (2002). Innovative Care for Chronic Conditions: Building blocks for Action Geneva.

[B2] Department of Health Improving chronic disease management. http://www.dh.gov.uk/assetRoot/04/07/52/13/04075213.pdf.

[B3] Murray CJ, Lopez AD (1997). Alternative projections of mortality and disability by cause 1990–2020: Global burden of disease study. Lancet.

[B4] van der Molen T, Price D (2000). The Founding of the International Primary Care Respiratory Group. Prim Care Respir J.

[B5] Holmes WF, Macfarlane J (1999). Issues at the interface between primary and secondary care in the management of common respiratory disease: Introduction. Thorax.

[B6] Department of Health (2000). The NHS Plan: a plan for investment, a plan for reform London.

[B7] Department of Health Implementing a scheme for GPs with Special Interests. http://www.dh.gov.uk/PolicyAndGuidance/OrganisationPolicy/PrimaryCare/GPsWithSpecialInterests.

[B8] Royal College of General Practitioners and Royal College of Physicians (2001). General Practitioners with special interest London.

[B9] Department of Health and Royal College of General Practitioners (2002). Implementing a scheme for General Practitioners with Special Interests London.

[B10] Williams S, Ryan D, Price D, Langley C, Fletcher M, Everden P (2002). General Practitioners with a special clinical interest: a model for improving respiratory disease management. Br J Gen Pract.

[B11] Gerada C, Wright N, Keen J (2002). The general practitioner with a special interest: new opportunities or the end of the generalist practitioner?. Br J Gen Pract.

[B12] Lung and Asthma Information Agency (1996). Factsheet 3 Respiratory morbidity in General Practice 1971–1991 London.

[B13] British Thoracic Society (2001). The Burden of lung disease London.

[B14] Damiani M, Dixon J (2002). Managing the pressure Emergency Hospital admissions in London 1997–2001.

[B15] Price D, Duerden M (2003). Chronic obstructive pulmonary disease – the lack of a national service framework should not allow us to ignore it. BMJ.

[B16] European Respiratory Society and European Lung Foundation (2003). European Lung White Book Geneva.

[B17] Partridge MR (2001). The profile of respiratory conditions: why government action is necessary. Thorax.

[B18] Department of Health Guidelines for the appointment of General Practitioners with Special Interests in the delivery on clinical services: respiratory medicine. http://www.dh.gov.uk/PolicyAndGuidance/OrganisationPolicy/PrimaryCare/GPsWithSpecialInterests.

[B19] The Respiratory Alliance Bridging the Gap. http://www.gpiag.org/news/bridging.php.

[B20] Royal College of Physicians (2003). Containing the Allergy Epidemic London.

[B21] Boynton PM, Greenhalgh T (2004). Selecting, designing and developing your questionnaire. BMJ.

[B22] Bryman A (2001). Qualitative data analysis. Social Research Methods.

[B23] Department of Health National Service Frameworks. http://www.dh.gov.uk/PolicyAndGuidance/HealthAndSocialCareTopics/HealthAndSocialCareArticle/fs/en?CONTENT_ID=4070951&chk=W3ar/W.

[B24] NHS Confederation, British Medical Association (2003). New GMS contract 2003: investing in general practice London.

[B25] National Primary Care Development Trust Primary Care Collaborative Phase III: Improving Chronic Disease Management.

[B26] Tashakkori A, Teddlie C (1998). Mixed Methodology: Combining Qualitative and Quantitative Approaches.

[B27] Nocon A, Leese B (2004). The role of UK general practitioners with special interests: implications for policy and service delivery. Br J Gen Pract.

[B28] Department of Health (2002). Advice on capacity plans for waiting, booking & choice London.

[B29] Sanderson D, on behalf of York Health Economics consortium Evaluation of the GPwSI pilot projects within the Action on ENT programme: Final Report.

[B30] Department of Health (2001). The expert patient: a new approach to chronic disease management for the 21st century London.

[B31] House of Commons Health Committee (2004). HC 696-I The Provision of Allergy Services.

[B32] Gruffydd-Jones K, for the General Practice Airways Group, British Thoracic Society, Royal College of Nursing, National Respiratory Training Centre, Respiratory Education and Training Centre (2005). A proposal for the accreditation of GPwSIs in respiratory medicine. Prim Care Respir J.

[B33] Rosen R, Stevens R, Jones R (2003). General Practitioners with special clinical interests: A potentially valuable asset, which requires evaluation. BMJ.

[B34] Rafferty S, Elborn JS (2002). Do nurses do it better?. Thorax.

[B35] Jones RCM, Freegard S, Reeves M, Hanney K, Dobbs F (2001). The role of the practice nurse in the management of asthma. Prim Care Respir J.

[B36] Taylor SJ, Candy B, Griffiths CJ, Wedzicha JW, Shirn B, Bryar R, Ramsay J, on behalf of the COPD nurse innovations review group (2003). Respiratory Nurse Specialist Interventions for patients with COPD in the Community: extended systematic review and extent of provision [abstract]. Thorax.

[B37] Ram FSF, Wedzicha JA, Wright J, Greenstone M (2004). Hospital at home for patients with acute exacerbations of chronic obstructive pulmonary disease: systematic review of evidence. BMJ.

